# Methyl 3,4-*O*-isopropyl­idene-2-*O*-[(methyl­sulfan­yl)thio­carbon­yl]-β-l-arabinoside

**DOI:** 10.1107/S1600536808011458

**Published:** 2008-04-30

**Authors:** Hongqi Li, Yanxi Song, Xiumei Li

**Affiliations:** a Key Laboratory of the Science and Technology of Eco-Textiles, Ministry of Education, College of Chemistry, Chemical Engineering and Biotechnology, Donghua University, Shanghai 201620, People’s Republic of China; b College of Environmental Science and Engineering, Donghua University, Shanghai 201620, People’s Republic of China; c Department of Physics and Technology, Inner Mongolia Tongliao Vocational College, Tongliao 028000, People’s Republic of China

## Abstract

In the title compound, C_11_H_18_O_5_S_2_, the six- and five-membered rings adopt a chair and an approximately planar conformation, respectively.

## Related literature

For related literature, see: Zhang *et al.* (1999 ▶).
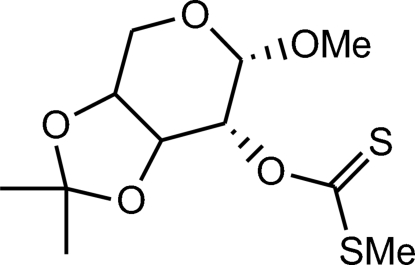


## Experimental

### 

#### Crystal data

C_11_H_18_O_5_S_2_
*M* *_r_* = 294.37
Orthorhombic, 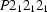

*a* = 9.1381 (9) Å
*b* = 11.2898 (11) Å
*c* = 13.9405 (14) Å
*V* = 1438.2 (2) Å^3^
*Z* = 4
Mo *K*α radiation
μ = 0.38 mm^−1^
*T* = 293 (2) K
0.50 × 0.41 × 0.39 mm

#### Data collection

Bruker APEX CCD diffractometer
Absorption correction: multi-scan (*SADABS*; Sheldrick, 2004 ▶) *T*_min_ = 0.752, *T*_max_ = 1.000 (expected range = 0.649–0.863)
8467 measured reflections
3115 independent reflections
2487 reflections with *I* > 2σ(*I*)
*R*_int_ = 0.098

#### Refinement

*R*[*F* ^2^ > 2σ(*F* ^2^)] = 0.042
*wR*(*F* ^2^) = 0.099
*S* = 0.95
3115 reflections
167 parameters
H-atom parameters constrained
Δρ_max_ = 0.25 e Å^−3^
Δρ_min_ = −0.19 e Å^−3^
Absolute structure: Flack (1983 ▶), 1306 Friedel pairs
Flack parameter: −0.05 (8)

### 

Data collection: *SMART* (Bruker, 2001 ▶); cell refinement: *SAINT* (Bruker, 2001 ▶); data reduction: *SAINT*; program(s) used to solve structure: *SHELXTL* (Sheldrick, 2008 ▶); program(s) used to refine structure: *SHELXTL*; molecular graphics: *SHELXTL* software used to prepare material for publication: *SHELXTL*.

## Supplementary Material

Crystal structure: contains datablocks I, global. DOI: 10.1107/S1600536808011458/wk2082sup1.cif

Structure factors: contains datablocks I. DOI: 10.1107/S1600536808011458/wk2082Isup2.hkl

Additional supplementary materials:  crystallographic information; 3D view; checkCIF report

## Figures and Tables

**Fig. 1.**
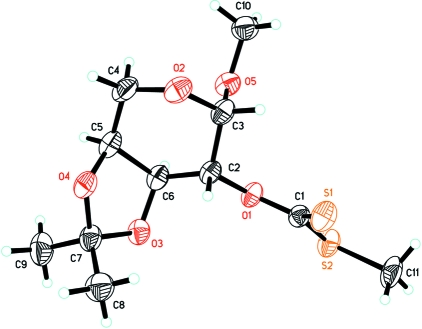
A view of the molecule of the title compound. Displacement ellipsoids are drawn at the 30% probability level and H atoms are shown as small spheres of arbitrary radii.

**Fig. 2.**
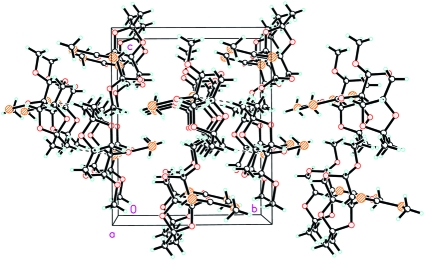
The crystal structure of the title compound, viewed along a axis.

## supplementary crystallographic information

### Comment

The title compound is an important intermediate for synthesis of 2-deoxy-*L*-ribose and was synthesized starting from *L*-arabinose according to the procedures reported by Zhang *et al.* (1999). Herein we present the single-crystal structure of methyl 3,4-*O*-isopropylidene-2-O– [(methylthio)thiocarbonyl]-β-*L*-arabinoside.

### Experimental

The title compound was prepared from *L*-arabinose according to the procedures reported by Zhang *et al.* (1999). Crystals suitable for X-ray diffraction were obtained by slow evaporation of a solution in ethyl acetate.

### Refinement

All H atoms were placed at calculated positions using a riding model, with C—H = 0.96Å for methyl C—H, 0.97Å for methylene C—H, and 0.98Å for methine C—H and with *U*_iso_(H) = 1.2*U*_eq_(C) except for methyl groups *U*_iso_(H) = 1.5*U*_eq_(C).

### Crystal data

**Table:**

C_11_H_18_O_5_S_2_	*D*_x_ = 1.360 Mg m^−^^3^
*M**_r_* = 294.37	Melting point = 403–400 K
Orthorhombic, *P*2_1_2_1_2_1_	Mo *K*α radiation λ = 0.71073 Å
*a* = 9.1381 (9) Å	Cell parameters from 3384 reflections
*b* = 11.2898 (11) Å	θ = 4.6–47.9º
*c* = 13.9405 (14) Å	µ = 0.38 mm^−^^1^
*V* = 1438.2 (2) Å^3^	*T* = 293 (2) K
*Z* = 4	Prismatic, colorless
*F*_000_ = 624	0.50 × 0.41 × 0.39 mm

### Data collection

**Table:**

Bruker APEX CCD diffractometer	3115 independent reflections
Radiation source: fine-focus sealed tube	2487 reflections with *I* > 2σ(*I*)
Monochromator: graphite	*R*_int_ = 0.098
*T* = 293(2) K	θ_max_ = 27.0º
φ scans	θ_min_ = 2.3º
Absorption correction: multi-scan(SADABS; Sheldrick, 2004)	*h* = −11→8
*T*_min_ = 0.752, *T*_max_ = 1.000	*k* = −14→14
8467 measured reflections	*l* = −17→17

### Refinement

**Table:**

Refinement on *F*^2^	Hydrogen site location: inferred from neighbouring sites
Least-squares matrix: full	H-atom parameters constrained
*R*[*F*^2^ > 2σ(*F*^2^)] = 0.042	*w* = 1/[σ^2^(*F*_o_^2^) + (0.0386*P*)^2^] where *P* = (*F*_o_^2^ + 2*F*_c_^2^)/3
*wR*(*F*^2^) = 0.099	(Δ/σ)_max_ = 0.001
*S* = 0.95	Δρ_max_ = 0.25 e Å^−^^3^
3115 reflections	Δρ_min_ = −0.19 e Å^−^^3^
167 parameters	Extinction correction: none
Primary atom site location: structure-invariant direct methods	Absolute structure: Flack (1983), 1306 Friedel pairs
Secondary atom site location: difference Fourier map	Flack parameter: −0.05 (8)

### Special details

**Table:**

Geometry. All e.s.d.'s (except the e.s.d. in the dihedral angle between two l.s. planes) are estimated using the full covariance matrix. The cell e.s.d.'s are taken into account individually in the estimation of e.s.d.'s in distances, angles and torsion angles; correlations between e.s.d.'s in cell parameters are only used when they are defined by crystal symmetry. An approximate (isotropic) treatment of cell e.s.d.'s is used for estimating e.s.d.'s involving l.s. planes.
Refinement. Refinement of *F*^2^ against ALL reflections. The weighted *R*-factor *wR* and goodness of fit *S* are based on *F*^2^, conventional *R*-factors *R* are based on *F*, with *F* set to zero for negative *F*^2^. The threshold expression of *F*^2^ > σ(*F*^2^) is used only for calculating *R*-factors(gt) *etc*. and is not relevant to the choice of reflections for refinement. *R*-factors based on *F*^2^ are statistically about twice as large as those based on *F*, and *R*- factors based on ALL data will be even larger.

### Fractional atomic coordinates and isotropic or equivalent isotropic displacement parameters (Å^2^)

**Table:**

	*x*	*y*	*z*	*U*_iso_*/*U*_eq_	
S1	0.98335 (7)	1.00851 (6)	0.84959 (5)	0.0646 (2)	
S2	0.91603 (8)	0.75403 (5)	0.89614 (5)	0.0669 (2)	
O1	0.72545 (17)	0.90443 (12)	0.86747 (11)	0.0509 (4)	
O2	0.5721 (2)	1.16759 (15)	0.75190 (13)	0.0639 (5)	
O3	0.51511 (18)	1.00383 (13)	1.00502 (12)	0.0567 (4)	
O4	0.44353 (18)	1.18675 (13)	0.95542 (11)	0.0593 (4)	
O5	0.5692 (2)	0.97000 (14)	0.70389 (12)	0.0605 (4)	
C1	0.8723 (3)	0.90034 (18)	0.86865 (16)	0.0473 (5)	
C2	0.6525 (2)	1.01781 (17)	0.85773 (15)	0.0457 (5)	
H2	0.7075	1.0776	0.8937	0.055*	
C3	0.6417 (3)	1.0560 (2)	0.7547 (2)	0.0544 (6)	
H3	0.7408	1.0643	0.7286	0.065*	
C4	0.4237 (3)	1.1616 (3)	0.78384 (19)	0.0674 (7)	
H4A	0.3675	1.1162	0.7376	0.081*	
H4B	0.3837	1.2411	0.7852	0.081*	
C5	0.4044 (3)	1.1071 (2)	0.88035 (19)	0.0601 (6)	
H5	0.3020	1.0833	0.8883	0.072*	
C6	0.5033 (2)	1.00210 (18)	0.90391 (17)	0.0522 (5)	
H6	0.4582	0.9280	0.8824	0.063*	
C7	0.4676 (3)	1.1180 (2)	1.03915 (17)	0.0576 (6)	
C8	0.5872 (3)	1.1738 (2)	1.0974 (2)	0.0746 (8)	
H8A	0.6729	1.1831	1.0584	0.112*	
H8B	0.6097	1.1240	1.1513	0.112*	
H8C	0.5555	1.2499	1.1200	0.112*	
C9	0.3288 (3)	1.1016 (3)	1.0968 (2)	0.0898 (10)	
H9A	0.2923	1.1776	1.1163	0.115*	
H9B	0.3494	1.0546	1.1525	0.115*	
H9C	0.2567	1.0624	1.0581	0.115*	
C10	0.5657 (3)	0.9914 (3)	0.60277 (18)	0.0719 (7)	
H10A	0.4948	1.0517	0.5889	0.108*	
H10B	0.5395	0.9198	0.5699	0.108*	
H10C	0.6605	1.0171	0.5816	0.108*	
C11	1.1125 (3)	0.7582 (3)	0.8980 (2)	0.0867 (10)	
H11A	1.1483	0.7774	0.8351	0.110*	
H11B	1.1496	0.6822	0.9170	0.110*	
H11C	1.1446	0.8173	0.9428	0.110*	

### Atomic displacement parameters (Å^2^)

**Table:**

	*U*^11^	*U*^22^	*U*^33^	*U*^12^	*U*^13^	*U*^23^
S1	0.0495 (3)	0.0544 (3)	0.0899 (5)	−0.0063 (3)	−0.0028 (3)	0.0012 (3)
S2	0.0618 (4)	0.0451 (3)	0.0938 (5)	0.0156 (3)	0.0026 (4)	0.0094 (4)
O1	0.0464 (8)	0.0338 (7)	0.0726 (10)	0.0039 (6)	−0.0027 (8)	0.0054 (7)
O2	0.0733 (12)	0.0430 (8)	0.0754 (11)	0.0076 (9)	−0.0087 (10)	0.0124 (8)
O3	0.0616 (10)	0.0418 (8)	0.0667 (9)	0.0057 (9)	0.0069 (8)	0.0086 (7)
O4	0.0588 (10)	0.0438 (8)	0.0753 (10)	0.0135 (8)	−0.0043 (9)	0.0031 (8)
O5	0.0642 (11)	0.0504 (8)	0.0668 (10)	0.0004 (8)	−0.0101 (8)	−0.0006 (8)
C1	0.0475 (12)	0.0417 (11)	0.0526 (13)	0.0062 (9)	−0.0033 (10)	−0.0031 (10)
C2	0.0447 (11)	0.0299 (9)	0.0626 (14)	0.0015 (9)	−0.0038 (10)	0.0039 (10)
C3	0.0484 (13)	0.0420 (11)	0.0726 (16)	0.0007 (11)	−0.0045 (11)	0.0051 (11)
C4	0.0679 (17)	0.0574 (14)	0.0767 (18)	0.0202 (15)	−0.0196 (15)	0.0030 (13)
C5	0.0428 (12)	0.0521 (12)	0.0854 (18)	0.0067 (11)	−0.0077 (13)	0.0011 (13)
C6	0.0465 (12)	0.0382 (10)	0.0719 (15)	−0.0009 (11)	−0.0028 (11)	0.0009 (11)
C7	0.0583 (16)	0.0469 (12)	0.0676 (15)	0.0092 (11)	0.0070 (13)	0.0079 (11)
C8	0.0845 (19)	0.0596 (15)	0.0799 (18)	0.0064 (16)	−0.0079 (17)	−0.0018 (14)
C9	0.070 (2)	0.095 (2)	0.104 (2)	0.0147 (18)	0.0279 (18)	0.005 (2)
C10	0.0788 (18)	0.0741 (17)	0.0629 (15)	0.0076 (16)	−0.0103 (15)	−0.0025 (14)
C11	0.0576 (16)	0.085 (2)	0.117 (2)	0.0362 (16)	0.0070 (16)	0.017 (2)

### Geometric parameters (Å, °)

**Table:**

S1—C1	1.610 (2)	C4—H4B	0.9700
S2—C1	1.742 (2)	C5—C6	1.526 (3)
S2—C11	1.796 (3)	C5—H5	0.9800
O1—C1	1.343 (3)	C6—H6	0.9800
O1—C2	1.450 (2)	C7—C8	1.501 (4)
O2—C3	1.412 (3)	C7—C9	1.513 (4)
O2—C4	1.429 (3)	C8—H8A	0.9600
O3—C6	1.414 (3)	C8—H8B	0.9600
O3—C7	1.441 (3)	C8—H8C	0.9600
O4—C7	1.419 (3)	C9—H9A	0.9600
O4—C5	1.426 (3)	C9—H9B	0.9600
O5—C3	1.372 (3)	C9—H9C	0.9600
O5—C10	1.431 (3)	C10—H10A	0.9600
C2—C3	1.503 (3)	C10—H10B	0.9600
C2—C6	1.518 (3)	C10—H10C	0.9600
C2—H2	0.9800	C11—H11A	0.9600
C3—H3	0.9800	C11—H11B	0.9600
C4—C5	1.490 (4)	C11—H11C	0.9600
C4—H4A	0.9700		
			
C1—S2—C11	101.97 (14)	C2—C6—C5	110.47 (18)
C1—O1—C2	119.41 (16)	O3—C6—H6	110.4
C3—O2—C4	112.1 (2)	C2—C6—H6	110.4
C6—O3—C7	108.58 (16)	C5—C6—H6	110.4
C7—O4—C5	107.30 (18)	O4—C7—O3	105.34 (18)
C3—O5—C10	113.5 (2)	O4—C7—C8	109.2 (2)
O1—C1—S1	127.00 (16)	O3—C7—C8	109.6 (2)
O1—C1—S2	105.33 (15)	O4—C7—C9	111.9 (2)
S1—C1—S2	127.66 (14)	O3—C7—C9	108.6 (2)
O1—C2—C3	111.91 (17)	C8—C7—C9	112.0 (2)
O1—C2—C6	105.68 (16)	C7—C8—H8A	109.5
C3—C2—C6	112.33 (18)	C7—C8—H8B	109.5
O1—C2—H2	108.9	H8A—C8—H8B	109.5
C3—C2—H2	108.9	C7—C8—H8C	109.5
C6—C2—H2	108.9	H8A—C8—H8C	109.5
O5—C3—O2	113.57 (19)	H8B—C8—H8C	109.5
O5—C3—C2	108.75 (18)	C7—C9—H9A	109.5
O2—C3—C2	108.2 (2)	C7—C9—H9B	109.5
O5—C3—H3	108.8	H9A—C9—H9B	109.5
O2—C3—H3	108.8	C7—C9—H9C	109.5
C2—C3—H3	108.8	H9A—C9—H9C	109.5
O2—C4—C5	114.4 (2)	H9B—C9—H9C	109.5
O2—C4—H4A	108.7	O5—C10—H10A	109.5
C5—C4—H4A	108.7	O5—C10—H10B	109.5
O2—C4—H4B	108.7	H10A—C10—H10B	109.5
C5—C4—H4B	108.7	O5—C10—H10C	109.5
H4A—C4—H4B	107.6	H10A—C10—H10C	109.5
O4—C5—C4	111.9 (2)	H10B—C10—H10C	109.5
O4—C5—C6	100.59 (17)	S2—C11—H11A	109.5
C4—C5—C6	116.4 (2)	S2—C11—H11B	109.5
O4—C5—H5	109.2	H11A—C11—H11B	109.5
C4—C5—H5	109.2	S2—C11—H11C	109.5
C6—C5—H5	109.2	H11A—C11—H11C	109.5
O3—C6—C2	110.65 (18)	H11B—C11—H11C	109.5
O3—C6—C5	104.42 (18)		
			
C2—O1—C1—S1	−6.7 (3)	O2—C4—C5—C6	−38.1 (3)
C2—O1—C1—S2	172.78 (14)	C7—O3—C6—C2	102.62 (19)
C11—S2—C1—O1	−179.09 (17)	C7—O3—C6—C5	−16.2 (2)
C11—S2—C1—S1	0.4 (2)	O1—C2—C6—O3	76.4 (2)
C1—O1—C2—C3	83.2 (2)	C3—C2—C6—O3	−161.29 (17)
C1—O1—C2—C6	−154.27 (19)	O1—C2—C6—C5	−168.44 (17)
C10—O5—C3—O2	65.6 (3)	C3—C2—C6—C5	−46.1 (2)
C10—O5—C3—C2	−173.94 (19)	O4—C5—C6—O3	32.2 (2)
C4—O2—C3—O5	55.4 (3)	C4—C5—C6—O3	153.3 (2)
C4—O2—C3—C2	−65.4 (2)	O4—C5—C6—C2	−86.8 (2)
O1—C2—C3—O5	57.3 (2)	C4—C5—C6—C2	34.3 (3)
C6—C2—C3—O5	−61.4 (2)	C5—O4—C7—O3	28.4 (2)
O1—C2—C3—O2	−178.93 (17)	C5—O4—C7—C8	146.0 (2)
C6—C2—C3—O2	62.4 (2)	C5—O4—C7—C9	−89.5 (3)
C3—O2—C4—C5	54.0 (3)	C6—O3—C7—O4	−6.3 (2)
C7—O4—C5—C4	−161.4 (2)	C6—O3—C7—C8	−123.6 (2)
C7—O4—C5—C6	−37.1 (2)	C6—O3—C7—C9	113.8 (2)
O2—C4—C5—O4	76.8 (3)		
